# The first review on prenatal drug exposure and ocular malformation occurrence

**DOI:** 10.3389/fped.2024.1379875

**Published:** 2024-09-04

**Authors:** Charlotte Dubucs, Julie Plaisancié, Monique Courtade-Saidi, Christine Damase-Michel

**Affiliations:** ^1^ Département d'Anatomie et Cytologie Pathologiques, Institut Universitaire du Cancer de Toulouse Oncopole, Toulouse, France; ^2^ Department of Medical and Clinical Pharmacology, University Hospital Center, CERPOP INSERM UMR 1295 – SPHERE Team, Toulouse, France; ^3^ Service de Génétique Médicale, Centre Hospitalier Universitaire de Toulouse, Toulouse, France

**Keywords:** ocular birth defect, prenatal medication exposure, congenital eye malformation, pharmacology, ophthalmopediatrics, microphthalmia, coloboma, cataracts

## Abstract

Even though a non-negligible portion of congenital eye anomalies has a clear genetic origin, an etiology is not found for most patients. Prenatal medication exposure is recognized to be involved in fetal malformations and several medications are specifically known to alter eye morphogenesis during embryonic development leading to congenital eye defects. We explored and reviewed the role of medications described in the genesis of ocular malformations, a role that has been little evaluated and probably still underestimated especially since several studies have shown the wide exposure of pregnant women to medication. We present our results in two sections; the first describes medications reported to be associated with ocular malformations in humans; the second details medications responsible for ocular malformations in animal models. We have summarized these results in tables, providing a relevant tool for clinicians. As most of the associations between medication exposure and congenital eye defects are either old or single case reports, this study highlights the needs for high epidemiological vigilance, accurate clinical description as well as a combination of studies on human genetics and experimental studies. Since medication exposures are potentially modifiable risk factors for congenital anomalies, this represents an important opportunity to implement preventive measures.

## 1 Introduction

Congenital eye anomalies are among the most common causes of visual impairment in children living in developed countries (1). They result from defects during the different stages of ocular morphogenesis which is a complex process requiring multiple cellular and genetic interactions in the early embryo. The organization of a three-dimensional ocular structure is an extremely conserved process across the vertebrate species that requires the interactions of multiple tissues with different embryonic origins (2): neuroectoderm, surface ectoderm and neural crest cells which will colonise the peri-ocular mesenchyme. The neuroectoderm gives rise to the following components of the eye: retina, posterior iris and optic nerve. The surface ectoderm induces the lens and corneal epithelium. Neural crest cells contribute to the corneal stroma and endothelium, the ciliary body, the uveal stroma and melanocytes, the sclera, meningeal sheaths and connective tissue of the optic nerve (3). Ocular development starts as early as week three of gestation with the appearance of optic grooves from the neuroectoderm in the developing forebrain (4). As the neural folds fuse, the optic grooves evaginate to form the optic vesicle. The optic vesicles protrude through the surrounding mesenchyme towards the surface ectoderm and lead to both the creation of the optic stalk, the future optic nerve and induce lens placode formation from the surface ectoderm. The lens placode invaginates towards the optic vesicle and produces the lens vesicle that individualizes from the outer surface ectoderm layer. At four weeks gestation, the optic vesicle invaginates, creating the optic cup and participating in future retina formation. A transient fissure forms along the ventral surface of the optic cup, called the choroid fissure, to allow the passage of the hyaloid vessels that become framed inside the optic nerve at the time of fissure closure. The proximal hyaloid vessels become the central retinal artery and vein while the distal hyaloid vessels, that supplied the developing lens, degenerate into the vitreous body (5). The fissure will close during week seven of gestation. As a result, the eye develops very early with all its structures in place before the 8th week of gestation.

Developmental defects may occur at any stage of the developing eye. Congenital abnormalities may affect any part of the eye and can be divided into three groups depending on the anatomic structure affected in the developing eye (6) (Table 1): (i) abnormalities of the anterior segment of the eye when it involves this particular part of the eye which includes the cornea, iris, ciliary body and lens; (ii) eyeball growth and formation defects: anophthalmia and microphthalmia corresponding to small or absent eye(s) respectively, and coloboma when a failure in fissure closure occurs) and (iii) posterior segment dysgeneses such as retinal dysplasia, abnormalities of the optic nerve or persistent hyperplastic primary vitreous (where there is a failure of resorption in the distal hyaloid vessels). According to different studies, the prevalence of congenital eye anomalies is estimated to be 2.4 and 7.5 per 10,000 births (7–9). Different causes are known: environmental, infectious [TORCH: Toxoplasmosis, Others (e.g., syphilis, HIV), Rubella, Cytomegalovirus (CMV) and Herpes simplex virus), toxic (alcohol, tobacco, medications) and genetic. After having excluded acquired causes, genetic investigations are usually conducted in patients. Some ocular phenotypes, especially when bilateral and/or severe, have commonly a genetic origin [i.e., up to 80% of genetic diagnoses in patients with bilateral anophthalmia (10), up to 90% with aniridia (11)] but the genetic origin is less obvious for some phenotypes, representing for example only 15% in patients with coloboma (12). These ocular phenotypes can sometimes be integrated into well-defined genetic syndromes such as CHARGE syndrome (Coloboma, Heart defect, Atresia choanae, Retarded growth, Genital hypoplasia and Ear abnormalities/deafness) PDAC (Pulmonary hypoplasia, Diaphragmatic hernia, Anophthalmia, Cardiac defect) or Renal-Coloboma syndrome (13). Among the emblematic genes implicated in ocular morphogenesis and whose mutations cause ocular malformations, we can quote *PAX6* (MIM*607108) as the “master” gene in eye development whose mutations are mainly linked to a panocular disorder called aniridia; *SHH* (MIM*600725) responsible for the division of the eye field into two optic vesicles for which pathogenic variants are associated with cyclopia (holoprosencephaly spectrum); or *PAX2* (MIM*167409) which is essential for optic stalk formation and closure of the choroid fissure in which pathogenic variants are responsible for coloboma. However, the origin behind ocular malformations in some patients remains unsolved and the participation of medications in the genesis of such malformations is little studied in humans except for a few “old” medications, well-known for their polymalformative teratogenicity such as Thalidomide and Isotretinoin (14–18). We therefore believe that there is a significance to investigating the role of certain medications in the genesis of ocular malformations, a role that is perhaps still underestimated. Several medications taken during pregnancy have been reported to be associated with congenital ocular anomalies in humans (Mycophenolate Mofetil, Methotrexate, Misoprostol, Hydroxyethylrutoside, Tranylcypromine, Coumarin anticoagulants, Valproic acid…) (19–28) and around thirty medications have been described as being associated with congenital ocular anomalies in different animal models (such as mice, rats and zebrafish) (29–36). In addition, several studies have demonstrated the wide exposure of pregnant women to medications. For example, an average of 10 different medications are prescribed during pregnancy in France (37, 38). Moreover, the lack of pregnancy safety data despite the common use of medication in pregnancy is a fact (39). Since pregnant women are until now generally excluded from clinical trials, treatments commonly used in pregnancy are therefore often old and untested, not optimized in dose and prescribed off-label without adequate safety information. Furthermore, given the early timing of eye development, medication exposures may occur when the woman herself is unaware of her pregnancy (and the majority of pregnancies are unplanned or mistimed).

**Table 1:** Groups of ocular congenital anomalies according to the anatomic structure affected in the developing eye.

1	Abnormalities of the anterior segment of the eye	Involve the cornea, iris, ciliary body and the lens (such as Axenfeld-Rieger anomaly, Peters’ anomaly, cataract and aniridia)
2	Eyeball growth and formation defects	Anophthalmia
		Microphthalmia (size at birth <14 mm)
		Coloboma
3	Posterior segment dysgeneses	Retinal dysplasia
		Abnormalities of the optic nerve
		Persistent hyperplastic primary vitreous

Several worldwide studies have shown that many causes of blindness in children are either preventable or treatable and therefore avoidable (40). In light of new knowledge on the subject, as a starting point for further reflections and studies, we consequently undertook a scoping review that aims to explore and summarize in a single review all medications previously published as being associated with an ocular defect.

## 2 Materiel and methods

We conducted a scoping review following the methodological framework described by Munn et al. (41) in order to summarize scientific knowledge of *in utero* medication exposure and ocular malformation occurrence as well as identify major research gaps. We therefore performed an extensive literature review of studies conducted in the English language which examined the associations between non-selected medications and ocular birth defects in humans and animal models. We restricted our research to ocular congenital malformations (functional ophthalmic abnormalities such as reduced acuity, nystagmus, delayed visual maturation, strabismus, refractive errors or cerebral visual impairment were evoked only when associated within the reported eye malformation). The literature was identiﬁed from PUBMED through April 2023 by combining different terms including *eye, ocular, congenital, defects, cataracts, lens, anophthalmia, microphthalmia, aniridia, coloboma, cornea, optic nerve hypoplasia, birth, defect* for ocular birth defects and 4 terms for the use of medications during pregnancy (*pharmacology, drugs, pregnancy, medication*). We only included studies if they speciﬁed ocular birth defect subtypes and a link to a specific medication or medication category. We also carried out a “snowball” search by searching the reference lists of the full texts for additional studies and using Google Scholar to identify and screen studies citing them (42). Each medication selected with a possible connection to an ocular defect was then checked in Reprotox (https://reprotox.org), a database that contains summaries on the effects of medications, chemicals, biological and physical agents on pregnancy, reproduction, lactation and development in humans and other species. Coloboma, cataract and microphthalmia were also specific key words verified in Reprotox since they are the three main ocular malformations frequently linked to teratogenic medications.

## 3 Results

The results are presented into two sections. The first section describes medications previously reported to be associated with ocular malformations in humans. We divided this section into two paragraphs; the first reveals medications that have a proven teratogenic effect on eye development in humans whereas the second discusses medications with a potential teratogenic impact but for which not sufficient knowledge exists to state a confirmed effect on eye development. A second section details medications involved in ocular malformation in animal models where the potential teratogenic effect on eye development in humans has not been proven to date.

### 3.1 What is known in humans?

The principal findings are summarized in Table 2.

**Table 2.** Drugs previously associated with ocular malformation in humans from literature

Drugs with known teratogenic effect on eye development		
Medications	Ocular anomaly type	References
3.1.1.1 Thalidomide	Ocular motility disturbances with Moebius syndrome	(14, 15, 16, 28, 43, 44, 45)
	Coloboma	
	Anophthalmia/microphthalmia	
	Buphthalmos	
	Pigmentary retinopathy	
	Congenital glaucoma	
	Hypertelorism	
	Congenital cataract	
	Optic nerve anomaly (myelinated nerve fiber, optic nerve palsies)	
3.1.1.2 Vitamin A/ Isotretinoin/ Retinoic acid	Microphthalmia	(18, 22, 28, 46, 47, 48, 49)
	Hypertelorism	
	Optic nerve hypoplasia	
	Small orbit	
3.1.1.3 Antiepileptic drugs (AED): phenobarbital (PHB), Primidone (PMD), phenytoin (PHT), barbiturates, Valproate (VPA), Carbamazepine (CBZ), diazepam (DZP)		(19, 20, 22, 50, 51, 52, 53, 54, 55, 56, 57, 58, 59)
PHT	Hypertelorism	
VPA	Coloboma	
PHT, PHB, DZP, CBZ, VPA	Optic nerve hypoplasia	
3.1.1.4 Chemotherapeutics and immune-system suppressant		
Methotrexate	Proptotic eyes	(23, 60)
	Hypertelorism	
Mycophenolate mofetil	Coloboma	(21, 27, 61, 62, 63)
	Anophthalmia/microphthalmia	
	Hypertelorism	
Cyclophosphamide	Optic nerve hypoplasia	(64–73, 74, 75)
3.1.1.5 Misoprostol	Möbius syndrome	(24, 76–78, 79)
3.1.1.6 Coumarin anticoagulant	Congenital cataract	(28, 80)
	Hypertelorism	
	Optic atrophy	
	Optic nerve hypoplasia	
	Peters anomaly	
3.1.1.7 Methimazole	Coloboma	(35, 81–83)
Drugs with discussed or potential teratogenic effect on eye development		
Medications	Ocular anomaly type	References
3.1.2.1 Hydroxyethylrutoside	Coloboma	(25)
3.1.2.2 Opioid		
Methadone	Optic nerve atropy	(84, 85)
	Nystagmus	
	Delayed visual maturation	
	Strabismus	
Opioid analgesics	Congenital glaucoma	(86)
	Anterior chamber eye defects	
3.1.2.3 Vitamins		
Pyridoxine	Congenital cataract	(87)
Vitamin D	Congenital cataract	(88)
3.1.2.4 Hormonal drugs		
*Corticoids*		
Inhaled corticoesteroids	Congenital cataract	(89)
Cortisone	Congenital cataract	(90, 91)
	Cyclopia	
*Thyroid hormone*		
Thyroxine	Congenital cataract	(92, 93)
3.1.2.5 Antihypertensive Angiotensin converting enzyme (ACE) inhibitors	Congenital glaucoma	(94)
	Coloboma	(95)
3.1.2.6 Antibacterial drug		
Nitrofurantoine	Anophthalmia/microphthalmia	(96)
Ethambutol	Anophthalmia/microphthalmia	(97, 98, 99)
3.1.2.7 Nonsteroidal antiinflammatory drug		
Aspirin, Ibuprofen, Naproxen	Anophthalmia/microphthalmia	(100)
3.1.2.8 General Anesthetics	Congenital cataract	(101–104)
3.1.2.9 Dalteparin	Congenital cataract	(105)
3.1.2.10 Quinine	Optic nerve hypoplasia	(106–109)
	Anophthalmia/microphthalmia	
	Retinal degeneration	
	Peters anomaly	
3.1.2.11 Allopurinol	Microphthalmia	(110)
3.1.2.12 Benzodiazepine: Alprazolam	Anophthalmia/microphthalmia	(111)
3.1.2.13 Chemotherapy: Carmustine	Microphthalmia	(112)

#### 3.1.1 Medications with known teratogenic effect on eye development

Indications and mechanisms of action of the medications discussed in this paragraph are available in Supplementary Table S1.

##### 3.1.1.1 Thalidomide

Thalidomide is a very strong teratogen capable of causing severe systemic malformations. Several babies of mothers who had taken this particular medication early in pregnancy were born with ocular defects. A survey by the Chief Medical Officer from the Ministry of Health of Great Britain relating to children born with congenital limb deformities during 1960–1962 revealed that there were 244 surviving children with such deformities whose mothers had taken Thalidomide during pregnancy (14). Among them, 27 presented additional deformities, two-thirds of which affected either eyes or ears or both. The colobomatous defect seems a typical eye deformity to be expected in Thalidomide children (43). Indeed, iris and choroid coloboma had been found as frequently as 25% among babies *in utero* exposed to Thalidomide in an old study from 1967 (16). These colobomas, which are caused by failure of choroid fissure closure during embryonic life, are easily diagnosed when the iris part of the eye is involved. There can be all kinds of severities ranging from a simple iris coloboma to lesions involving the ciliary body, retina, choroid or optic nerve. Later, a review conducted in 1991 on ocular teratogenic agents reported additional eye anomalies such as microphthalmia, anophthalmia and lens anomalies (28). In a study of 100 Swedes manifesting thalidomide embryopathy, Miller et al. also described buphthalmos (15). A review dating from 1999 demonstrated Thalidomide involvement in additional ocular signs including strabismus, aberrant lacrimation, glaucoma, lipodermoid of conjunctiva, hypertelorism, myelinated nerve fiber as well as ptosis (44). The large number of affected children worldwide exposed to Thalidomide, and the many informative cases where the exposure time was known, made it possible to build a teratogenic timetable relating affected structures to the time of exposure. Miller et al. showed that Thalidomide had a teratogenic effect between 20 and 36 days after fertilization (45). Incidentally, they found that Duane syndrome (abnormal innervation of extra-ocular muscles leading to congenital strabismus) was prominent in individuals who were exposed to Thalidomide early on in the sensitive period (days 20–26). However, structural eye malformations were less frequent in this early phase, appearing slightly later during the sensitive period. The precise details of thalidomide's teratogenic mechanism are complex and may involve a combination of factors; angiogenesis inhibition: Thalidomide's anti-angiogenic effects may disrupt this process, leading to malformation of developing limbs and other structures; interference with limb bud development: Thalidomide is thought to interfere with the outgrowth and differentiation of limb buds, resulting in limb abnormalities; oxidative stress: Thalidomide can induce oxidative stress, leading to damage to developing cells. Oxidative stress is known to play a role in teratogenicity, and thalidomide's impact on cellular processes may contribute to developmental abnormalities; binding to protein targets: Thalidomide is known to bind to specific proteins, including cereblon. Cereblon is a substrate receptor of the E3 ubiquitin ligase complex, which plays a role in protein degradation. By binding to cereblon, Thalidomide alter its function and disrupts the normal ubiquitination and subsequent degradation of certain target proteins, leading to various downstream effects such as teratogenic effect.

##### 3.1.1.2 Vitamin A/Isotretinoin/retinoic acid

In 2009, Tandon et al. discussed the fact that both hypo- and hypervitaminosis A have been known to be connected with fetal developmental defects (22). Vitamin A is especially needed for eye formation during pregnancy and by the retina for both low-light and color vision during adulthood (46, 47). Isotretinoin is a vitamin A synthetic analogue used for treatment of severe acne. Its use during pregnancy which causes high levels of vitamin A is teratogenic and linked to multiple fetal malformations known as *retinoic acid embryopathy*. Eye anomalies have previously been observed in retinoic acid embryopathy such as microphthalmia, optic nerve hypoplasia and small orbit (18, 28, 48). On the other hand, retinol deficiency is known to occur when pathogenic variants affect the gene encoding for its blood transporter, the serum retinol binding protein *RBP4* (MIM*180250). Pathogenic variants in this gene lead to two distinct phenotypes that involve ocular malformations; microphthalmia with coloboma (MIM#616428) and retinal dystrophy with coloboma and comedogenic acne syndrome (MIM#615147). The disturbance of vitamin A homeostasis leads to neural crest cell disruption directly involving ocular development defect (49).

##### 3.1.1.3 Anti-epileptic medications (AED): phenobarbital (PHB), primidone, phenytoin (PHT), valproate (VPA), and carbamazepine (CBZ)

Many studies have assigned a teratogenic effect to AED, associating malformations, intrauterine or postnatal growth failure as well as psychomotor retardation depending on the type of medication (20). In 1999, a description of visual and ocular outcome in infants after prenatal exposure to AED showed no major eye anomalies except in one child who had nystagmus and low vision (50). The results therefore suggest that well-controlled treatments with AED during pregnancy do not have any major adverse effects on eye development and visual functions. However, contradictory findings appeared in different papers regarding prenatal CBZ exposure and the occurrence of congenital eye malformations. According to the authors, due to both low eye malformation prevalence and low frequency of CBZ exposure during pregnancy, it is complicated to eliminate an increased relative risk (51). Some studies identified additional risk factors, for example, elevated maternal serum AED concentrations, high daily AED dosage or low folate levels. Of late, several ocular defects have been well-described as part of “*fetal anticonvulsant syndrome”* (22). Although there are differences in phenotypes after exposure depending on the type of medication, a significant overlap of features exists. Description in a small series of children with prenatal VPA exposure suggested a high incidence of early onset myopia and strabismus (52). In addition, other studies also reported coloboma occurrence in VPA exposed infants (53, 54). Recently (55), the term “*Fetal Valproate Spectrum Disorder”* (FVSD), now well-known in the medical community, was proposed by the expert consensus. Another anticonvulsant known as Trimethadione, most commonly used to treat resistant epilepsy, has been linked to “*fetal trimethadione syndrome.”* This condition includes developmental delay with speech difficulty, palatal anomaly, abnormal teeth and in some patients, ocular anomalies such as strabismus and myopia but no real ocular malformation (19). Hoyt and Billson found cases of optic nerve hypoplasia associated with prenatal exposure to phenytoin, phenobarbital and diazepam (56). Two case reports of *in utero* exposure to carbamazepine and valproic acid with optic nerve hypoplasia/dysplasia were also reported (57, 58). The teratogenicity mechanism is probably different depending on AED type, for example it is believe that VPA induce selective apoptosis of ocular fibrous tunic with collagen deficiency in corneal stroma and sclera/choroid layers (59).

##### 3.1.1.4 Chemotherapeutics and immune-system suppressants

###### 3.1.1.4.1 Methotrexate

Methotrexate is a chemotherapy agent and immune-system suppressant. It is used to treat cancer, autoimmune diseases (such as psoriasis, rheumatoid arthritis and Crohn's disease) and ectopic pregnancy. It is also used in conjunction with misoprostol to induce medical abortion. Both of these medications are well-known teratogens and patients who were exposed to these medications during the first trimester of pregnancy exhibited a significant teratogenic risk to develop polymalformative anomalies. Their specific ocular teratogenic effects have been associated to proptotic eyes and ocular hypertelorism (23). It has been presumed that excessive cell death for which the embryo may be unable to compensate was responsible of the eye defect (60).

###### 3.1.1.4.2 Mycophenolate mofetil

Mycophenolate mofetil (MMF) is an immunosuppressant medication used to prevent rejection following organ transplantation and to treat many autoimmune diseases such as Crohn's disease, rheumatoid arthritis and lupus nephritis (21, 61). Ocular anomalies such as iris or chorioretinal coloboma and anophthalmia/microphthalmia have been associated to *in utero* MMF exposure (21, 27). They are part of a well-recognized “*MMF teratogenic syndrome*” in association with external ear malformation, cleft lip and palate, congenital heart defects, distal limbs anomalies, vertebral malformations, esophageal atresia, diaphragmatic hernia, kidney and central nervous system anomalies. A case report also cited hypertelorism in association with ocular coloboma, esophageal atresia and microtia (21, 62). MMF, inhibits inosine monophosphate dehydrogenase (IMPDH), an enzyme involved in the *de novo* synthesis of guanosine nucleotides (63). By inhibiting this enzyme, MMF disrupts the proliferation of T and B lymphocytes, which are key components of the immune system. During embryonic development, cells undergo rapid division and differentiation, and there is a high demand for DNA synthesis. The inhibition of IMPDH by MMF may interfere with the DNA synthesis required for normal embryonic development. This disruption in DNA synthesis is thought to contribute to the teratogenic effects of mycophenolate.

###### 3.1.1.4.3 Cyclophosphamide

Cyclophosphamide is an alkylating agent used in cancer and autoimmune diseases. Numerous studies have demonstrated that babies with cyclophosphamide embryopathy have intrauterine growth restriction, skeletal defects and craniofacial malformations with eye anomalies which include microphthalmia and hypoplasia of the optic nerve (64–73). The data concerning these possible effects are difficult to interpret since pregnant women exposed to this medication have serious diseases for which they are being treated and multiple pharmaceutical agents are usually used in conjunction with cyclophosphamide. Interestingly, Cyclophosphamide increased birth defects in all the experimental animal species tested. The following eye developmental effects among others were observed in the animal experiments: anophthalmia/microphthalmos, microphakia and aphakia, thinning and anterior segment dysgenesis, excessive hyaloid vasculature and ectopia lens (74, 75).

##### 3.1.1.5 Misoprostol

Several studies suggested a link between first trimester *in utero* Misoprostol exposure and Möebius syndrome occurrence (paralysis of facial and external eye muscles that limit facial expression) in their babies (24, 76–78). It has been previously suggested that Möebius syndrome may be due to subclavian artery vascular disruption during the fourth to sixth week of development. Misoprostol exposure during the first two months of pregnancy was thought to cause an ischemic event in the embryonic brain stem that results in Möebius syndrome (79). The subclavian artery is a major blood vessel that arises from the aorta and supplies blood to the arms and some structures in the head and neck. Vascular disruptions or abnormalities in the subclavian artery can potentially impact blood supply to various regions, including the structures developing from embryonic blood vessels. The embryo undergoes complex processes of blood vessel formation during development, and disruptions in blood flow can lead to congenital defects.

##### 3.1.1.6 Coumarin anticoagulants

In 1991, a review on presumed ocular teratogenic agents revealed that administration of oral anticoagulants causes congenital malformations known as “*warfarin embryopathy”* (28). Abnormalities included nasal hypoplasia, stippled epiphyses, developmental retardation in association with eye anomalies. Specific ocular anomalies included cataracts, small eyelids, hypertelorism, optic atrophy and optic nerve hypoplasia. Kaplan et al. also reported Peters anomaly associated with Dandy-Walker syndrome and agenesis of the corpus callosum but with none of the signs of warfarin embryopathy (80). During embryonic development, vitamin K is essential for the synthesis of certain proteins involved in bone and cartilage formation. These proteins are known as vitamin K-dependent proteins. The inhibition of vitamin K by warfarin can affect the synthesis of these proteins, potentially leading to warfarin embryopathy.

##### 3.1.1.7 Methimazole

While Methimazole (MTI) is widely used to treat maternal hyperthyroidism during pregnancy, different studies have reported that *in utero* exposure to MTI results in a large spectrum of congenital anomalies including ocular malformations such as iris and retinal coloboma (81–83). These results were recently supported by animal studies where the authors confirmed the teratogenic effects of MTI on the development of zebrafish and provided experimental evidence for the connection between exposure to MTI and human MTI embryopathy (35). The developing fetus relies on maternal thyroid hormones, especially during the early stages of pregnancy when the fetal thyroid gland is not yet fully functional. Methimazole, by inhibiting thyroid peroxidase, interferes with the synthesis of thyroid hormones, specifically triiodothyronine (T3) and thyroxine (T4). The thyroid hormones are crucial for normal fetal development, particularly for the development of the central nervous system and skeletal structures. When thyroid hormone synthesis is disrupted, it can lead to congenital hypothyroidism in the fetus.

#### 3.1.2 Medications with discussed or potential teratogenic effect on eye development

These medications are treated in a separate section due to the limited amount of data available on them. Indeed, the majority of associations observed in studies discussed in this section are as yet unconﬁrmed. For most medications, the numbers of exposed infants were too small to draw conclusions about their teratogenic risks. Indications and mechanisms of action of medications discussed in this paragraph are summarized in Supplementary Table S1. Most of the time, no information is yet available on a hypothesis of teratogenicity mechanism.

##### 3.1.2.1 Hydroxyethylrutoside

Although Hydroxyethylrutoside (HER) is frequently utilized in pregnant women for the treatment of vascular diseases, it has been correlated with teratogenic effects (25). In 2013, Pósfai et al. discovered that oral HER treatment during early pregnancy is related to a higher risk for unilateral ocular coloboma [association of HER treatment during the second and/or third month of pregnancy showed an Odds Ratio (OR) with 95% CI: 5.4, 2.2–12.9]. This study was made in the population-based Hungarian Case-Control Surveillance System of Congenital Abnormalities, including 22,843 cases with congenital abnormalities, 567 (2.5%) had mothers with HER treatment while of 38,151 matched controls, 1143 (3.0%) were born to mothers with HER treatment (OR with 95% CI: 0.8, 0.7–0.9).

##### 3.1.2.2 Opioids

###### 3.1.2.2.1 Methadone

Prescribed for daily use, Methadone relieves cravings and removes withdrawal symptoms. In 2010, Hamilton et al. described a case series of 20 patients exposed to substitute methadone *in utero*, all of whom were referred to a pediatric visual electrophysiology unit because of visual function difficulties (84). Ophthalmic abnormalities included nystagmus, reduced acuity, delayed visual maturation, strabismus, refractive errors and cerebral visual impairment but no real underlying ocular malformation. However, in another older study from 2005, malformations occurred in 12 of 78 newborns *in utero* exposed to Methadone and comprised one case of optic nerve atrophy (85).

###### 3.1.2.2.2 Opioid analgesics

A report from 2011 using data from the National Birth Defects Prevention Study (NBDPS) database (a large population-based case-control study of major birth defects in the United States) found statistically significant positive associations between maternal use of opioid analgesics and primary congenital glaucoma or anterior chamber eye defects (adjusted OR, 2.6; 95% CI, 1.0–6.6) (86). However, the study only included 5 exposed cases.

##### 3.1.2.3 Vitamins

###### 3.1.2.3.1 Pyridoxine

Recently, Schrager et al. used data from the case-control NBDPS database to examine whether first-trimester nausea and vomiting during pregnancy or its specific treatments were associated with 37 major birth defects (87). Among the 37 births defects analysed, cataract was the only ocular anomaly that was considered in the study, and authors observed an increased risk of cataracts (2.57, 1.12–5.88).

###### 3.1.2.3.2 Vitamin D

During pregnancy, calcitriol is made in the fetoplacental unit. The ability of the fetus to produce calcitriol is believed to play a role in the fetal control of placental calcium transfer. Only one report of prenatal vitamin D deficiency associated with cataracts in a child was published. However, vitamin D deficiency and cataracts occurrence was not demonstrated in a controlled study from 1996 (88). On the other hand, excess vitamin D has not been linked to a syndrome of congenital anomalies.

##### 3.1.2.4 Hormonal medications

###### 3.1.2.4.1 Corticoids

####### 3.1.2.4.1.1 Inhaled steroids

In 2016, Garne et al. reported a meta-analysis from three cohort studies (519,242 pregnancies in Norway, Wales and Funen in Denmark) to examine the effect of maternal exposure to asthma medications on the risk of congenital anomalies (89). The authors found a link between congenital cataracts and exposure to inhaled steroids. Although this association was based on five cases from Wales with no exposed cases in Norway and Denmark, the authors found an odds ratio of 3.53 (1.05–11.81). Given the limited number of exposure occurrences, other analysis with more cases must be undertaken.

####### 3.1.2.4.1.2 Cortisone

In 1985, Schardein et al. described 35 infants with first trimester exposure to cortisone, with congenital defects detected in nine of them (90). The malformations included cataracts and cyclopia but also interventricular septal defect, gastroschisis, hydrocephalus, cleft lip, coarctation of the aorta, clubfoot and undescended testis without an evident pattern of defects. Congenital cataracts were also reported in one infant exposed to prednisone throughout the pregnancy (91).

###### 3.1.2.4.2 Thyroid hormone

####### 3.1.2.4.2.1 Thyroxine

There have been reports of human infants with malformations including ocular defects born after maternal treatment with thyroid hormones although most reports featured the concomitant use of other medications, often including antithyroid medications (92). Animal teratogenicity studies associate thyroxine with adverse effects on lens development in rats (93).

##### 3.1.2.5 Antihypertensive medications

A study conducted by Forestieri et al. investigated potential risk factors for primary congenital glaucoma (PCG) in the NBDPS database. Incidentally, the authors found associations with all cases of PCG and antihypertensive use by mothers [adjusted odds ratio (AOR) = 3.60, CI 1.52–8.53] (94). Maternal antihypertensive use was also associated with isolated PCG (AOR 3.55, 95% CI 1.39–9.10). However, antihypertensive medication type is not specified in the study. Since these particular medications have very heterogeneous mechanisms of action, it is difficult to draw conclusions about the potential association.

###### 3.1.2.5.1 Angiotensin converting enzyme inhibitors

Angiotensin converting enzyme (ACE) inhibitors are associated with fetal damage and death during the second and third trimesters of pregnancy. An epidemiological study published in 2006 reported an increased risk of cardiovascular and central nervous system (CNS) malformations in infants whose mothers received a prescription for an ACE inhibitor during the first trimester (95). Among the three cases grouped as having CNS defects, the authors included 1 infant with spina bifida, 1 with microcephaly and an undefined “eye anomaly” and a third with coloboma. The statistical analysis was based on a population of 209 mothers with assumed exposure to an ACE inhibitor. The authors indicated the small study population to be a limitation of their findings and a high body mass index as a possible confounded result.

##### 3.1.2.6 Antibacterial medications

###### 3.1.2.6.1 Nitrofurantoin

Crider et al. evaluated the correlation between selected birth defects and antibacterial medication use during early pregnancy (96). Interestingly, the authors found that Nitrofurantoins were associated with anophthalmia or microphthalmia (with AOR = 3.7; 95% CI, 1.1–12.2). However, caution must be exercised since there were only 4 exposed cases.

In this same report, the authors found statistically significant positive links between the use of any antibacterial medications and primary congenital glaucoma/other anterior segment dysgenesis defects (96). This association lost its significance when looking at the different specific antibacterial medications separately.

###### 3.1.2.6.2 Ethambutol

One case report relates to a woman treated with ethambutol, rifampin and isoniazid during the first trimester who gave birth to a child with microphthalmia, other malformations of one eye and absence of the other eye (97). However, case reports are not informative on causation and to date, the avoidance of ethambutol in initial therapy is based on the possibility of optic neuritis in the offspring given this complication can be associated with ethambutol in adults (98). Such a complication has not been observed in neonates although the lack of reports might be due to the difficulty of detecting optic neuritis in a newborn (98). According to the product labeling, ethambutol treatment in pregnant rats has been associated with two fetuses with monophthalmia and two fetuses with extra ocular anomalies (one fetus with a shortened right forelimb and bilateral wrist-joint contracture while the other had a cleft lip and palate) (99).

##### 3.1.2.7 Nonsteroidal anti-inflammatory medications and aspirin

Hernandez et al. analyzed whether the use of nonsteroidal anti-inflammatory medications (NSAID) in early pregnancy was related to a range of structural birth defects using data from the NBDPS database (100). The authors found increased risks of anophthalmia/microphthalmia associated with Ibuprofen [AOR = 1.9 (1.1–3.3)], Aspirin [AOR = 3.0 (1.3–7.3)] and Naproxen [AOR = 2.8 (1.1–7.3)] exposure respectively (100). This study is important due to the large incidence of anophthalmia/microphthalmia numbering 123 cases of which 8, 19 and 7 cases were exposed to Aspirin, Ibuprofen and Naproxen respectively. However, the association between anophthalmia/microphthalmia and NSAIDs was restricted to cases with accompanying defects and no significant causal relationship was found with isolated anophthalmia/microphthalmia, raising the question of a possible alternative etiology. It is worthwhile to specify that out of the 34 NSAID-exposed anophthalmia/microphthalmia cases, 7 had unilateral craniofacial anomalies that were suggestive of possible hemifacial macrosomia according to the authors.

##### 3.1.2.8 General anesthetics

Exposure to anesthetics during gestation occurs in 2% of all pregnant women as a result of emergency or elective surgery (113, 114). Moreover, anesthesia can be administered during gestation for fetal procedures. In 5,405 cases of single general anesthesia exposure in early pregnancy, there was no correlation with birth defects (101, 102). Maternal recall of general anesthesia during the 1st trimester was associated with hydrocephalus and eye defects (primarily cataracts) in 12 births (103, 104).

##### 3.1.2.9 Dalteparin

On the basis of experimental animal studies and poor placental passage in the second and third trimesters, Dalteparin use during pregnancy is not expected to increase the risk of birth defects. Even if no conclusions can be drawn from a single case, a randomized multinational open-label trial reported that six infants exposed to Dalteparin exhibited congenital anomalies, including one with cataracts and the other five with extra-ocular malformations (two with ankyloglossia, one with ectopic kidney, one with trisomy, one with strawberry hemangioma) (105).

##### 3.1.2.10 Quinine

###### 3.1.2.10.1 Chloroquine & hydroxychloroquine

In animal models, Chloroquine administration to pregnant mice and monkeys results in transplacental transfer as well as accumulation in the fetal adrenal cortex and retina (115, 116). Chloroquine produced anophthalmia and microphthalmia in rat fetuses after treatment of the dams at very high doses (>250 mg/kg) (117). Recently, experiments from *in vitro* and *in vivo* models showed that chloroquine generated genetic mutations and chromosomal damage (summary of product characteristics of Nivaquine®). Interestingly, two old cases of retinal degeneration after chloroquine *in utero* exposure were also published (106). Hydroxychloroquine, like Chloroquine, crosses the placenta and the volume of distribution of these two medications is very high. Moreover, both their half-lives are long: 10–30 days for chloroquine and 30–60 days for hydroxychloroquine which leads to prolonged exposure. Therefore, a woman who takes one of these medications prior to a pregnancy can potentially be exposed during a new pregnancy (107). Ocular adverse effects have been described with both chloroquine and hydroxychloroquine in treated patients such as retinal damage, sometimes irreversible, after several years of exposure. The ocular toxicity of these medications is dose-dependent as well as contingent on the duration of medication use (108). Since these medications can induce severe ocular adverse effects, a risk for the fetus cannot be excluded. To date, one single case of Peters anomaly was described in a child *in utero* exposed to methotrexate and hydroxychloroquine (109).

##### 3.1.2.11 Allopurinol

Hoeltzenbein et al. reported 31 women who used allopurinol during pregnancy including the first trimester (110). The authors found a prematurity rate of 19%, possibly due to maternal disease. All but two of these women were also taking other medications. Of the 5 infants with congenital anomalies, four had minor malformations and one was born with microphthalmia in association with cleft lip and palate, renal hypoplasia, low-set ears, hearing deficiency, bilateral cryptorchidism and micropenis (110). However, no causality can be made since this concerns one case and the mother of this child was also taking other medications (hydrochlorothiazide, pyridoxine and sodium carbonate) and a genetic cause cannot be excluded.

##### 3.1.2.12 Benzodiazepine

###### 3.1.2.12.1 Alprazolam

One case-control study reported an association between alprazolam use in early pregnancy and anophthalmia/microphthalmia based on 3 exposed cases (OR 4.0, 95% CI 1.2–13.1) (111). There was no adjustment for confounders, including other benzodiazepines or multiple comparisons.

##### 3.1.2.13 Cancer chemotherapy

###### 3.1.2.13.1 Carmustine

A single case reports a woman who used Carmustine with other chemotherapy agents (Dartmouth combination chemotherapy) during her first and second trimester and delivered a child diagnosed with isolated microphthalmia and hypermetropia aged 1 year (112).

### 3.2 What is known in animal models?

For the previously mentioned medications, several animal studies have already been well-described and published (i.e., chicks, mice, pigs, zebrafishes) and these researches into animal studies has contributed significantly to understanding the pathophysiology of these well-known teratogens (22, 35, 118–122). Apart from these described medications, other medications are still only known to be associated with congenital eye anomalies in animal models. We reviewed them in this paragraph and the principal findings are summarized in Table 3. Indications and mechanisms of action of the medications discussed in this paragraph are summarized in Supplementary Table S2. We only considered observations at non-maternotoxic doses and medications such as Levocabastine, Oxybutynin, Tolvaptan known to lead to ocular malformations in the presence of maternal toxicity were not reported.

**Table 3:** Drugs previously associated with ocular malformation in animal models.

Medication	Ocular anomaly type	References
3.2.1 Anti-cancer agents	** **	
3.2.1.1 Chemotherapy	** **	
Paclitaxel	Congenital cataract	(123, 124)
	Retinal dysplasia	
5-Fluorouracil (5-FU)	Microphthalmia	(125)
Cisplatin	Microphthalmia	(126, 127)
Hydroxyurea (hydroxycarbamide; Hydrea)	Anophthalmia/microphthalmia	(128)
Chloraminophene	Optic nerve atrophy	(30)
	Coloboma	
	Congenital cataract	
	Microphthalmia	
2-chloro-2'-deoxyadenosine (2-CdA)	Microphthalmia	(32, 129, 130)
Leflunomide/teriflunomide	Anophthalmia/microphthalmia	(131–133)
Encorafenib	Microphthalmia	(134, 135)
3.2.1.2 Alkylating agent		
Busulfan	Microphthalmia	(136)
Melphalan	Anophthalmia/microphthalmia	(137)
3.2.1.3 angiogenic inhibitors (TNP-470 and Angiostatin 4.5)	Microphthalmia	(34)
3.2.2 Azilsartan	Congenital cataract	(138)
3.2.3 Treatment of Parkinson disease		
Levodopa	Ocular suppurative inflammation	(139, 140)
	Congenital cataract	
Entacapone	Macrophthalmia	(141)
	Anophthalmia/microphthalmia	
3.2.4 Glucagon	Congenital cataract	(142)
3.2.5 Norepinephrine (noradrenaline)	Congenital cataract	(143)
3.2.6 Dexrazoxane	Anophthalmia/microphthalmia	(144, 145)
3.2.7 Anti-infectious		
Efavirenz	Anophthalmia/microphthalmia	(146, 147)
Rifapentine	Microphthalmia	(148, 149)
Oteseconazole	Exophthalmos	(150–152)
	Congenital cataract	
	Optic nerve atrophy	
	Ocular haemorrage	

#### 3.2.1 Anti-cancer drugs

##### 3.2.1.1 Chemotherapy

###### 3.2.1.1.1 Paclitaxel

Cataract and retinal dysplasia developed in neonatal rats treated with paclitaxel 4 mg/kg administered intraperitoneally (123). Intravenous treatment of pregnant rats eight days after coitus with a clinical paclitaxel preparation produced embryofetal death and malformation at a maternal dose level of 2 mg/kg (124).

###### 3.2.1.1.2 5-Fluorouracil

According to Reprotox, malformations have occurred with 5-FU in all the species tested. In mice, 5-FU caused multiples anomalies including microphthalmia (125).

###### 3.2.1.1.3 Cisplatin

In chicks, microphthalmia was associated with a single-dose cisplatin injection administered to the embryos where it appeared in 88% of the surviving embryos (126). The decrease in eye size was noticeable 2- or 3-days post-injection. The authors found that macroscopic, histological as well as ultrastructural changes started to appear in the ciliary body and suggested that microphthalmia was the consequence of decreased pressure (127).

###### 3.2.1.1.4 Hydroxyurea

Hydroxyurea produced malformations in experimental animal pregnancies in chicks, rats, mice, cats and monkeys. Defects involved the central nervous system, eyes, face, and limbs and more specifically, ano/microphthalmia in Wistar, SD and Fisher rat fetuses (128).

###### 3.2.1.1.5 Chloraminophene/Chlorambucil

In mice, Chloraminophene directly induces ocular abnormalities (30). The injection of 200 µg of Chloraminophene into pregnant mice has a strong teratogenous effect. If the injection is given on the 9th day then the optic nerve is absent, the choroid fissure persists on the 10th day while lens are abnormal and the size of the cups is reduced on days 11, 12, 13 and 14. The normal organization of the secondary lens fibers is disturbed by the destruction of part of the primary fibers.

###### 3.2.1.1.6 2-Chloro-2'-deoxyadenosine

Although there are cases of normal outcome after human pregnancy exposure (129), 2-chloro-2'-deoxyadenosine (2-CdA) interferes with embryo development and viability in experimental rats. In these animals, gestational exposure is associated with microphthalmia, shortened trunk and lumbar hernia (130). In terms of ocular development, it has been shown that 2-CdA administered to pregnant mice induces microphthalmia through a mechanism linked to the p53 tumor suppressor pathway (32). Interestingly, PK11195, an isoquinoline carboxamide, capable of binding selectively to the peripheral benzodiazepine receptor, was able to suppress the pathogenesis of microphthalmia. This implies a critical role for mitochondrial peripheral benzodiazepine receptors in the p53-dependent mode of action of 2-CdA on eye development.

###### 3.2.1.1.7 Leflunomide

The product labelling contains a warning against the use of leflunomide during pregnancy based on experimental animal studies where administration of 15 mg/kg/day in pregnant rats increased congenital malformations in the offspring (131). Malformations included anophthalmia/microphthalmia and hydrocephalus. This dose level was maternally toxic and caused embryolethality as well as decreased fetal weight among survivors. The 15 mg/kg/day dose level in pregnant rats produced plasma concentrations over time that were 1/10 those achieved in humans on therapy. Treatment of pregnant rabbits with 10 mg/kg/day increased fused and dysplastic sternebrae in the offspring. Based on plasma concentrations, this dose level was equivalent to human dosing. No uptick in congenital anomalies occurred in rats or rabbits at the maternal dose level of 1 mg/kg/day.

###### 3.2.1.1.8 Teriflunomide

Experimental animal findings were summarized in the product labelling (132) and the FDA reviewer report but these two conclusions differed (133). According to the product labelling, oral dose levels of 1, 3, or 10 mg/kg/day given to pregnant rats resulted in a high prevalence of fetal malformations and embryofetal death in the absence of maternal toxicity. By contrast, the FDA reviewer report stated that there was decreased maternal body weight in all treatment groups. Fetal malformations at the high dose level included axial and appendicular skeletal defects, hydrocephalus, anophthalmia and microphthalmia. Anophthalmia and microphthalmia associated with maternal toxicity can be seen in rats. The 1 mg/kg/day dose level produced maternal plasma concentrations below what is observed in humans at the maximum recommended dose of 14 mg/day. As per product labelling, pregnant rabbits administered this agent at oral dose levels of 1, 3.5, or 12 mg/kg/day exhibited fetal malformations and embryofetal death at dose levels linked with minimal maternal toxicity.

###### 3.2.1.1.9 Encorafenib

In line with the product labelling (134) and information from the US FDA non-clinical review (135), Encorafenib is associated with fetal anasarca, micrognathia and microphthalmia development in rats at 26 times the recommended human exposure dose which produced maternal toxicity.

##### 3.2.1.2 Alkylating agents

###### 3.2.1.2.1 Busulfan

Busulfan exposure causes microencephaly and microphthalmia in the offspring of pregnant rats given 10 mg/kg intraperitoneally (136). However, this administration is not relevant to human exposure.

###### 3.2.1.2.2 Melphalan

Product labelling stated that melphalan was embryolethal and teratogenic in rats with oral and intraperitoneal administration (137). Anomalies included malformations of the brain and eyes (anophthalmia and microphthalmia) as well as reduction of the mandible and tail and exomphalos.

##### 3.2.1.3 Anti-angiogenic medications

Authors have previously showed that the maternal use of angiogenic inhibitors (TNP-470 and Angiostatin 4.5) cause fetal growth restriction and placental abnormalities (34). During a rapid growth phase of ocular development (embryonic days 12–19) in mice, the placenta mediates the metabolic requirements of the fetus and therefore may impact the growth of ocular development during the highly oxygen sensitive phase. Angiogenic inhibitors can thereby induce microphthalmia either indirectly by their known effects on placental morphology and function or directly by blocking microvascular growth in the fetus. We could then hypothesize that any angiogenic inhibitor is capable of causing microphthalmia.

#### 3.2.2 Azilsartan

The use of Azilsartan is avoided in human pregnancy based on experience with other medications that interfere with the renin-angiotensin system. Experimental animal studies are summarized on the FDA website (138). Its use during pregnancy increases malformations in rabbits at maternally toxic dose levels. At 50 mg/kg/day, the authors found higher incidences of cataracts, thoracoschisis and persistent truncus arteriosus in the offspring. However, rats exhibited no increased malformations at maternal dose levels of up to 1,000 mg/kg/day in spite of maternal toxicity.

#### 3.2.3 Treatment of Parkinson's disease

##### 3.2.3.1 Levodopa

Even though to our knowledge, no reports in humans have identified abnormal embryo or fetal development, Levodopa is involved in adverse pregnancy outcomes in experimental animals (139). Indeed, Levodopa and dopamine are associated with early postnatal loss in rats in conjunction with various malformations including ocular defects such as cataracts and suppurative inflammation of the eyes (140).

##### 3.2.3.2 Entacapone

Female mice treated with 160 mg/kg/day entacapone (equivalent to 7 times the human dose on a plasma concentration basis), prior to mating and in early gestation, produced offspring with increased macrophthalmia, microphthalmia and anophthalmia (141).

#### 3.2.4 Glucagon

The use of glucagon to treat insulin-induced hypoglycemia is not expected to increase the risk of adverse pregnancy outcome. However, different eye defects such as cataracts have been observed in rat fetuses from mothers treated with glucagon during early pregnancy (142).

#### 3.2.5 Norepinephrine

Treatment of near-term rat fetuses with norepinephrine 0.025 mg/kg produced cataracts (143). The vasoconstrictive effects of norepinephrine raised the possibility that developmental effects associated with treatment were due to ischemia.

#### 3.2.6 Dexrazoxane

Developmental studies summarized in the product labelling reported that dexrazoxane treatment used in pregnant rats at 8 mg/kg/day (10% of the human dose on a surface area basis) produced an increased level of malformations in offspring including microphthalmia and anophthalmia (144). Maternal toxicity occurred at 2 mg/kg/day and the authors highlighted the fact that occurrence of anophthalmia and microphthalmia in rat pups might reflect maternal toxicity rather than constitute a direct effect of the medication on embryo development (145). Indeed, a wide range of other anomalies were found to be associated when malformations did occur and often within the same animal.

#### 3.2.7 Anti-infectious medications

##### 3.2.7.1 Efavirenz

Reproductive information is presented in the FDA review of this product (146). Three out of 20 cynomolgus monkey fetuses were born with congenital anomalies after maternal treatment with 60 mg/day (147). These abnormalities included anophthalmia and anencephaly in the first fetus, microphthalmia in the second fetus and cleft palate in the third fetus. At this dose level, plasma concentrations of the medication were similar to those obtained in human patients. Interestingly, placental transfer of efavirenz in monkeys is similar to that produced in humans. However, as this medication was included as first-line treatment for HIV there is a substantial amount of data on safety from real world studies – e.g., in a large European cohort collaboration there were no ocular defects reported in the EFV-exposed pregnancies (147). Detailed information on exposure to antiretrovirals, including EFV, is also available from the Antiretroviral Pregnancy Registry – again with no signal regarding eye defects associated with any drug (http://www.apregistry.com).

##### 3.2.7.2 Rifapentine

There are reports of normal outcomes after rifapentine use during human pregnancy but no controlled studies exist for humans. Rifapentine increases adverse outcomes in rat and rabbit pregnancies (148, 149). Different malformations were observed in 4 rabbit fetuses including absence of ovaries, absent nose, bent foot and microphthalmia at dose levels lower than those used for humans.

##### 3.2.7.3 Oteseconazole

In rats, oteseconazole produced several ocular malformations including cataracts, exophthalmos, optic nerve atrophy and ocular bleeding at 3.5 times the human dose (150–152).

## 4 Discussion

This work represents the first overview of medications previously described to be associated with ocular defects. The medications known to be involved in congenital ocular defects have been listed in a table according to their nature (Table 4). Although this list is exhaustive, it is likely that many other medications can lead to ocular malformations and their role in the genesis of such anomalies is probably underestimated since their use in pregnant women is usually limited and potential teratogenic effects, if any, remain unknown. If the different stages of eye development are well-described, their underlying molecular mechanisms are only partially understood. Eye morphogenesis is extremely complex and involves a series of intricated cascading cellular events, precisely orchestrated by a set of molecular signals that switch genetic programs on and off. We can easily imagine that any interference with one of these signalling pathways can disrupt the development of the eye in a similar manner than pathogenic variants affecting the expression of genes involved in eye development. A medication can possibly lead to a specific pathogenic process depending on the embryonic stage at which it is administered, its duration and dose of exposure as well as genetic susceptibility (153–155). A review by Van Gelder et al. focused on the teratogenic mechanisms associated with a number of medications (156). Authors identified six teratogenic mechanisms related to medication use: folate antagonism, neural crest cell disruption, endocrine disruption, oxidative stress, specific receptor- or enzyme-mediated teratogenesis and vascular disruption (156). Malformations attributed to the process of vascular disruption have been well-described as a distinctive entity among the recognized aetiologies (157). It is believed that blood flow to a structure was altered after that structure had formed normally. The reduction in blood flow leads to hypoxia, endothelial cell damage, haemorrhage and tissue loss. After recovery, some structures can exhibit either tissue loss or structural abnormalities. It has been suggested that Möebius syndrome may be due to vascular disruption of the subclavian artery during early embryonic development as previously discussed (158). Indeed, cases of infants with Möebius syndrome were reported following maternal antenatal splenic rupture and hypotension with Misoprostol exposition. Reduced vascular supply can also be seen in medications with a vasoconstrictive effect such as norepinephrine which can have a possible developmental effect due to ischemia (143). Furthermore, this mechanism of vascular disruption could explain asymmetric or unilateral defect particularly frequent in colobomas and microphthalmia.

**Table 4:** Ocular malformation type and corresponding associated prenatal medications exposure in humans.

Abnormalities of the anterior segment of the eye	Associated prenatal medications exposure
Congenital cataract	Thalidomide, Coumarin anticoagulant, Pyridoxine, Vitamin D, Inhaled corticoesteroids, Cortisone, Thyroxine, General Anesthetics, Dalteparin
Peters’ anomaly	Coumarin anticoagulant, Quinine
Congenital glaucoma	Thalidomide, Opioid analgesics, Antihypertensive
Unspecified anterior chamber eye defects	Opioid analgesics
Eyeball growth and formation defects	Associated prenatal medications exposure
Anophthalmia/microphthalmia	Thalidomide, Isotretinoin, Mycophenolate mofetil, Nitrofurantoine, Ethambutol, Aspirin, Ibuprofen, Naproxen, Quinine, Allopurinol, Alprazolam, Carmustine
Coloboma	Thalidomide, Antiepileptic drugs, Mycophenolate mofetil, Methimazole, Hydroxyethylrutoside, Angiotensin converting enzyme (ACE) inhibitors
Posterior segment dysgeneses	Associated prenatal medications exposure
Retinal degeneration	Quinine
Optic nerve anomaly, hypoplasia/atrophy	Thalidomide, Isotretinoin, Antiepileptic drugs, Cyclophosphamide, Coumarin anticoagulant, Methadone, Quinine
Pigmentary retinopathy	Thalidomide

Moreover, specific pathways such as retinoic acid (RA) signalling are of particular interest in eye development. RA is a biologically active metabolite of vitamin A that serves as a signalling molecule which plays multiple roles during eye development. The link between maternal vitamin A deficiency and fetal ocular defects such as microphthalmia has been known for a long time (49). RA signalling is required for interactions between the optic vesicle and lens placode and supports normal development of the retina and the optic nerve through its involvement in the periocular mesenchyme derived from neural crest cells. RA coordinates these actions by regulating specific receptors activities, RARα/β/γ and RXRα/β/γ. Inactivation of RARα/β/γ receptors in the periocular mesenchyme stops anterior eye segment formation. A review by Cvekl et al. summarizes genetic knowledge on RA signalling (159). These findings are concordant with the broad spectrum of ocular malformations in RA embryopathy such as microphthalmia, optic nerve hypoplasia and coloboma in a similar way as pathogenic variants in *STRA6* (MIM*610745) or *RARB* (MIM*180220) genes, both of which are responsible of dysregulation in the RA pathway and therefore involved in syndromic microphthalmia (i.e., PDAC). Knowledge on genetic bases is fundamental to better understand the molecular aspects involved in the processes of teratogenesis with similar pathways of genes and protein dysfunction caused by specific drug exposure. These “phenocopies” are of particular interest as they not only provide a better understanding of the pathophysiological mechanisms of developmental abnormalities (of genetic or teratogenic origin) but may also open up new therapeutics. Defects of varying nature can occur at different levels throughout various molecular targets within the same pathway and yet lead to the same adverse response. For example, holoprosencephaly through altered Shh signalling can be observed with alkaloids that alter the post-translational modifications of the Shh protein; by agents that inhibit specialized enzymes in the cholesterol synthesis pathway consequently affecting the Shh modifications; or by mutations in genes encoding for those enzymes or the Shh protein itself. Each of these agents acts differently at the molecular level but operates on the same pathway by altering Shh function (160).

Previous studies have analysed the association between the use of antihypertensive medications and birth defects with conflicting results (94). According to the authors, it is probable that the findings observed in some studies are related to the underlying maternal hypertension rather than the medications, especially since the term “antihypertensive” covers a wide variety of pharmaceutical classes. In fact, this is the case with any chronic maternal disease and caution must be taken when interpreting any associations. For instance, many maternal diseases are well-known to be involved in fetal development anomalies such as maternal dysthyroidism or diabetes (161–163). Maternal diabete has several known adverse effects on embryogenesis as well as fetal development and can causes multiple congenital anomalies and secondary medical complications collectively referred to diabetic embryopathy (164). A diabetic mother is prone to 2–3 times more risk of having a baby affected by a birth defect than a nondiabetic mother. Likewise, maternal thyroid disease, which is common in women of childbearing age, is associated with significant impact on fetal development.

Many medications discussed in Section 3.1.2 have been described in association with congenital eye anomalies and must be interpreted with caution since numerous of the published cases are either old publications or single case reports which do not demonstrate causation since most of them have been described only once or in very few cases. Moreover, some of the associations found have been described in multiple medications prescribed together and another difficulty is our inability to differentiate the teratogenic effects of medications from those of other medications administered concomitantly in the situation of combination therapy. Animal experiments are of particular value since they are able to bypass the multiple extraneous variables inherent to human case reports. They allow us to specifically analyse the effects of one or combined medications on gametes, preimplantation embryos, organogenesis as well as their potential teratogenic mechanisms even though their extrapolation from one species to another remains delicate. The contribution of human/animal comparisons is of particular interest since researchers are able to model human diseases effectively and affordably in an animal model to help understand the molecular basis of a disease and to test medications *in vivo*. Due to the highly conserved nature of eye development, there is a great variety of vertebrate species that can be utilized to understand not only pathological mechanisms but also the mechanisms and processes under normal conditions (165).

All medications discussed in Section 3.2 have been linked with ocular defects in animal models even though no adverse effects have yet been observed in humans. As these medications are not commonly used in pregnant women, their potential teratogenic effects are still unknown in humans. Interestingly, the same medication can lead to different teratogenic effects in different animal models. This divergence of phenotypes regarding the same teratogen makes it possible to discover other parallel pathways that escape in one direction or another similarly to how pathogenic variants in one gene lead to different phenotypes in various animal models. For instance, biallelic mutations in the *B3GLCT* gene (MIM*610308) are responsible for Peters plus syndrome in humans, a severe congenital glycosylation disorder where patients have different anomalies that include eye malformation such as Peters' anomaly, disproportionate short stature, dysmorphic facial features and developmental delay although it only generates small sized craniofacial and skeletal defects in mice and normal development in zebrafish (166, 167). A possible compensated mechanism in zebrafish with complete *b3glct* deficiency has been suggested by authors to explain their normal development. A comparison can be made with prenatal Methimazole (MTI) exposure. Although *in utero* exposure of MTI in humans results in a variety of congenital anomalies including choanal and oesophageal atresia, iris and retinal coloboma as well as delayed neurodevelopment (81–83), MTI treatment of pregnant rats, mice and rabbits does not increase the incidence of malformations in the offspring (168, 169). More recently, MTI treatment of pregnant rhesus monkeys during late pregnancy induced intellectual disability-like behaviors in the progeniture (170). However, no clear nervous system malformations were described and review of the ocular pathology did not seem to be undertaken. On the other hand, observation of MTI-exposed zebrafish embryos showed delayed development and hypoplasia of the whole brain and spinal cord, narrowing of the pharynx and oesophagus, severe disruption of the retina and aberrant structure of the notochord (35). These malformations are similar to the congenital anomalies observed in children *in utero* exposed to MTI. In this example, zebrafish provided experimental evidence for the link between exposure to MTI and human MTI embryopathy not found with other animal models (rat, mice or monkeys). It is possible that in these 3 latter species, there are other parallel signalling pathways that compensate the teratogenic mechanism of MTI. Interestingly, MTI embryopathy seems to be a clear phenocopy of the CHARGE syndrome (MIM# 214800), a genetic disease related to pathogenic variants in the *CHD7* gene. It would be interesting to deepen our knowledge about the molecular mechanisms causing this phenotype with both MTI exposure and *CHD7* pathogenic variants.

Another interesting example is about thalidomide medication, which was not teratogenic in rats (171). This fact explains why, since then, regulations on reproductive toxicology have been modified to require testing on several species, one of which being not a rodent.

This work highlights the importance of health professionals reporting and recording ocular adverse events following prenatal exposure to drugs through Phase IV Pharmacovigilance and post-marketing, as well as the importance of systematically recording cases of malformations through malformations registries throughout the world. The value of TIS (Teratogen Information Services), monitoring of patients exposed to drugs during pregnancy and, finally, the value of pharmacoepidemiological studies to improve knowledge in the link between ocular malformations and prenatal drug exposure, alongside experimental studies (since there is a “gap” in the clinical trial and we are moving directly from observation in animals to real-life post-marketing).

## 5 Conclusion

Congenital ocular malformations are rare diseases and regular updates are required given the description of unknown side-effects caused by known and new medications to market. This literature review aims to give a report of the current state of knowledge on the subject. As most of the associations found in the literature between medication exposure and congenital eye defects are either old or single case reports, this study supports the need for large-scale studies with special attention placed on malformation registries. It highlights the need for epidemiological vigilance, precise clinical description as well as a combination of studies on human genetics and experimental studies. Given medication exposures are possible modifiable risk factors for congenital anomalies, this represents a great opportunity to provide prevention strategies. Beyond this crucial aspect and applications in clinical practice, this review provides additional bases for researchers to understand and elucidate signalling pathways involved in ocular morphogenesis by comparing molecular results obtained from drug and genetic studies.
