# Supplementary material for: The first review on prenatal drug exposure and ocular malformation occurrence
Source: Front Pediatr. 2024 Sep 4;12:1379875. doi: 10.3389/fped.2024.1379875 (PMC11408236; doi:10.3389/fped.2024.1379875)
Supplement: Supplementary file 1 [file Datasheet1.doc]

**Supplementary Table 1**: *Indications and mechanisms of action of medications discussed in humans (see Table 2).*

| **Medications** | **Indication and mechanisms of action** | **Potential mechanism of teratogenic effect** |
| --- | --- | --- |
| **Medications with known teratogenic effect on eye development** | |  |
| **1.1.1 Thalidomide** | Thalidomide was initially marketed as a sedative hypnotic drug (1, 2). Thalidomide is an anti-inflammatory, immunomodulatory, and antiangiogenic agent experiencing resurgence in the treatment of dermatological diseases. Thalidomide is FDA-approved for the treatment of erythema nodosum leprosum and EMA-aproved as a first-line treatment in patients over 65 years of age with untreated multiple myeloma or with a contraindication to high-dose chemotherapy in combination. Reports also suggest its efficacy in actinic prurigo, aphthous stomatitis, Behçet disease, Kaposi sarcoma, the cutaneous manifestations of lupus erythematosus, and prurigo nodularis and uremic prurigo. | Angiogenesis inhibition, induction of oxidative stress, Binding to protein targets such as cereblon |
| **1.1.2 Vitamin A/ Isotretinoin/ Retinoic acid** | Vitamin A is a group of unsaturated nutritional [organic compounds](about:blank). Vitamin A includes [retinol](about:blank), [retinal](about:blank), and several [provitamin](about:blank) A [carotenoids](about:blank) and is not only important for growth and development, but also for vision (3).  *Retinoids* comprise natural and synthetic compounds that exhibit vitamin A–like biological activity or bind to nuclear receptors for retin­oids. They are used in dermatological disorders such as acne, psoriasis and in cutaneous T-cell lymphoma and Kaposi sarcoma. | Disturbance of vitamin A homeostasis |
| **1.1.3 Antiepileptic medications (AED): phenobarbital (PHB), Primidone, phenytoin (PHT), Valproate (VPA), and Carbamazepine (CBZ)** | Treatment with Antiepileptic medications (AED) started in the beginning 1900, with *phenobarbital* (PHB), which was found to have anti-seizure activity and had been considered as the first-line treatment for many years. Medications similar to PHB such as Primidone was developed. In 1940, phenytoin (PHT) was found to be an effective drug in the epilepsy treatment.  *Carbamazepine* (CBZ) has been employed since the 1960s for the treatment of trigeminal neuralgia. It was initially approved in the U.S. for use as an antiseizure agent in 1974.  *Valproate* (VPA) is used in focal and generalized seizures. Its action appears to be mediated by a prolonged recovery of voltage-activated Na+ channels from inactivation. Another potential mechanism that may contribute to *valproate*’s anti­seizure actions involves metabolism of GABA. Valproic acid is also a potent inhibitor of histone deacetylase. Thus, some of its antiseizure activity may be due to its ability to modulate gene expression through this mechanism. | Induction of selective apoptosis |
| **1.1.4 Chemotherapeutics and**[**immune-system suppressant**](about:blank) | Chemotherapeutics are used to treat cancers. These agents target critical processes for cell division in cancer cells. | Excessive cell death, disruption in DNA synthesis |
| **1.1.5 Misoprostol** | Misoprostol is a synthetic analog of prostaglandin E1 that was first indicated to prevent and treat stomach and duodenal ulcers.  It acts on the smooth muscle fibers in the myometrium with uterotonic properties and is currently used to cause abortion, induce labor, and treat postpartum bleeding in case of poor contraction of the uterus. | Induction of ischemic event by disruptions in blood flow |
| 1.1.6 Coumarin | The coumarin anticoagulants lower the functional levels of multiple coagulation factors acting on vitamin K cycle. Therapeutic doses of warfarin or other Vitamin K antagonists inhibit vitamin K epoxide reductase. | Inhibition of vitamin K, induction of oxidative stress |
| **1.1.7 Methimazole** | Methimazole is an antithyroid medication used to treat hyperthyroidism. It inhibits the formation of thyroid hormones by interfer­ing with the incorporation of iodine into tyrosyl residues of thyroglob­ulin; it also inhibits the coupling of these iodotyrosyl residues to form iodothyronines. | Inhibition of thyroid peroxidase which interferes with the synthesis of thyroid hormones |
| **Medications with discussed or potential teratogenic effect on eye development** | |  |
| **1.2.1 Hydroxyethylrutoside** | Hydroxyethylrutoside (HER) is used to treat chronic venous insufficiency. HER acts on the microvascular endothelium to reduce hyperpermeability and edema. In patients, HER is expected to improve microvascular perfusion and microcirculation, and reduce erythrocyte aggregation (4). | Induction of oxidative stress, DNA damage |
| 1.2.2 Opioid | Methadone, is a synthetic opioid agonist used for chronic pain and opioid dependence (5, 6). | Induction of apoptosis, oxidative stress, placental insufficiency, hormonal disruption |
| **1.2.3 Vitamins** | -***Pyridoxine*** is a vitamin used to treat vitamin B6 deficiency and nausea during pregnancy as an antiemetic use.  -***Vitamin D*** is an essential nutrient playing an active role in Ca2+ homeostasis. The biological actions of vitamin D are mediated by the vitamin D receptor (VDR) a nuclear receptor. Vitamin D is the name applied to two related fat-soluble substances, vitamin D3 (cholecalciferol) and vitamin D2 (ergocalciferol). | ***Pyridoxine***  Disruption of neurotransmitter balance, interference with folate metabolism, induction of oxidative stress  ***Vitamin D***  Disruption of calcium and phosphorus balance |
| **1.2.4 Hormonal medications** | - ***Glucocorticoids*** have broad anti-inflammatory effects on multiple components of cellular immunity. They bind to receptors inside cells on glucocorticoid response elements in DNA and regulate the transcription of genes. There are numerous therapeutic indications for glucocorticoids. They commonly are combined with other immunosuppressive agents to pre­vent and treat transplant rejection. Glucocorticoids also are efficacious for treatment of graft-versus-host disease in bone marrow transplantation. Glucocorticoids are routinely used to treat autoimmune disorders such as rheumatoid and other arthritis, systemic lupus eryth­ematosus, systemic dermatomyositis, psoriasis and other skin conditions, asthma and other allergic disorders, inflammatory bowel disease, inflam­matory ophthalmic diseases, autoimmune haematological disorders. In addition, glucocor­ticoids limit allergic reactions.  -***Thyroxine*** (T4), the major hormone produced by the thyroid, is a pro-hormone that is converted to triiodothyronine (T3). Thyroid hormones regulate many physiological processes such as growth and development, thermogenesis, cardiovascular system and lipid metabolism. In the bloodstream, thyroxine is predominantly bound to proteins and crosses the placenta poorly at physiologic concentrations. Levothyroxine is the hormone of choice for thyroid hormone replacement therapy due to its consistent potency and prolonged duration of action. | ***Glucocorticoids***  Inhibition of cell proliferation, induction of apoptosis, alteration of gene expression, induction of oxidative stress, inhibition of angiogenesis, Reduction of blood flow  ***Thyroxine***  Regulation of gene expression (Thyroxine regulates gene expression through thyroid hormone receptors (TRs) that bind to thyroid response elements (TREs) in the DNA, modulating the transcription of various target genes), metabolic imbalance (Thyroxine regulates basal metabolic rate and influences the metabolism of carbohydrates, fats, and proteins) |
| **1.2.5 Antihypertensive** | Antihypertensive drugs concern medications that are used to treat arterial  [hypertension](https://en.wikipedia.org/wiki/Hypertension). Antihypertensive drugs can be classified according to their sites or mechanisms of action. Drugs may decrease the cardiac output by inhibiting myocardial contractility or by decreasing ventricular filling pressure. Reduction in ventricular filling pressure may be achieved by actions on the venous tone or on blood volume via renal effects. Drugs can decrease peripheral resistance by acting on smooth muscle to cause relaxation of resistance vessels or by interfering with the activity of systems that produce constriction of resistance vessels (e.g., the sympathetic nervous system, the renin-angiotensin system). Low­ering of body sodium content (by diuretics) can indirectly lower peripheral resistance. | Blood pressure reduction |
| **1.2.6 Antibacterial drug** | - ***Nitrofurantoin*** is an antibacterial of the nitrofuran family that is used for the prevention and treatment of urinary tract infections. It is activated by enzymatic reduction, with the formation of highly reactive intermediates that seem to be responsible for the observed capacity of the drug to damage bacterial DNA. Nitrofurantoin is active against many strains of E. coli and enterococci.  ***- Ethambutol*** is an antituberculous drug. It inhibits arabinosyl transferase III, thereby disrupting the transfer of arabinose into arabinogalactan biosyn­thesis, which in turn disrupts assembly of the mycobacterial cell wall. | -***Nitrofurantoin*** Production of Reactive Oxygen Species (ROS) and oxidative stress, DNA damage (Nitrofurantoin can intercalate into DNA)  ***- Ethambutol***  Disruption of ribosome function, induction of oxidative stress |
| **1.2.7 Nonsteroidal anti-inflammatory medications and Aspirin** | -*Nonsteroidal anti-inflammatory drugs* (NSAIDs), are widely used as analgesics, antipyretics, and anti-inflammatory agents.  -*Low-dose aspirin* irreversibly inhibits platelet cyclooxygenase-1 (COX-1) and suppresses platelet aggregation. It is effective for secondary prevention of cardiovascular events.  The NSAIDs are mechanistically classified as *isoform nonselective NSAIDs*, which inhibit both COX-1 and COX-2, and *COX-2–selective NSAIDs*. Most NSAIDs are competitive, non-competitive, or mixed reversible inhibitors of the COX enzymes. The principal therapeutic effects of NSAIDs derive from their ability to inhibit prostaglandin production. | Inhibition of COX enzymes leading to prostaglandin inhibition (important for regulation of blood flow to the fetus and maintenance of the ductus arteriosus) |
| **1.2.8 General Anesthetics** | General anesthetics depress the central nervous system to a sufficient degree to permit the performance of surgery and unpleasant procedures. The leading theory of the molecular and cellular mechanisms by which general anesthetics produce their effects was that anesthesia is produced by perturbation of the physical properties of cell membranes. | Unknown |
| **1.2.9 Dalteparin** | Dalteparin is a low molecular weight heparin (LMWH). It binds to antithrombin and accelerate the rate at which it inhibits various coagulation proteases. LMWH, can be used to ini­tiate treatment of deep vein thrombosis and pulmonary embolism. They also can be used for the initial management of patients with unstable angina or acute myocardial infarction. | Unknown |
| **1.2.10. Quinine** | ***-Quinine*** is a cinchona alkaloid traditionally used to treat chloroquine-resistant malaria and leg cramps.  ***-Chloroquine*** is a quinine derivative that was developed as an antimalarial agent. Its anti-inflammatory properties at higher doses are used for lupus erythematosus and rheumatoid arthritis.  ***-Hydroxychloroquine*** (Plaquenil) is a quinine derivative, more potent than chloroquine (7). | DNA intercalation and damage, disruption of calcium homeostasis, induction of oxidative stress, impaired protein synthesis and function by its binding to ribosomes, induction of apoptosis |
| **1.2.11 Allopurinol** | Allopurinol is a hypoxanthine analog used in the treatment of recurrent calcium kidney stones, hyperuricemia, and gout. Allopurinol has been used in some patients with inflammatory bowel disease. Allopurinol works by inhibiting xanthine oxidase and decreasing the production of uric acid. | Induction of apoptosis and DNA damage, oxidative stress, alterations in cellular signaling pathways |
| **1.2.12 Benzodiazepine** | Alprazolam is a benzodiazepine, used as a tranquilizer and sedative. The anxiolytic effects are mediated by allosteric interactions with the pen­tameric benzodiazepine-GABAA receptor complex. The primary effect of the anxiolytic benzodiazepines is to enhance the inhibitory effects of the neurotransmitter GABA. | Interaction with GABA_A receptors, induction of apoptosis, induction of oxidative stress |
| **1.2.13 Cancer chemotherapy** | Carmustine (BiCNU; BCNU) is a nitrosourea used in cancer chemotherapy. It exerts its cytotoxicity through the spontaneous breakdown to an alkylating intermediate. DNA cross-linking appears to be the primary lesion responsible for the cytotoxicity of nitrosoureas. | DNA Alkylation and cross-linking, induction of apoptosis, induction of oxidative stress, disruption of cell signaling pathways |

**Supplementary Table 2**. *Indications and mechanisms of action of medications discussed in animals.*

| **Medications** | **Indication and mechanisms of action** | **Teratogenic effect suspected** |
| --- | --- | --- |
| **2.1 Anti-cancer agents** | |  |
| **2.1.1 Chemotherapy** | ***-Paclitaxel*** is an antineoplastic that prevents the depolymerization of tubulin, resulting in unrestricted accumulation of microtubules within cells. Microtubules are important in cell locomotion, transport, and secretion, and provide the framework on which chromatids separate in mitosis. Paclitaxel has been used as a coating on some coronary stents to inhibit coronary narrowing.  ***-5-Fluorouracil (5-FU)*** is a pyrimidine antineoplastic agent either injectable or topical cream.  ***- Cisplatin*** has been used as an antineoplastic agent.  ***- Hydroxyurea*** is an antineoplastic drug that reduces the synthesis of DNA. It has been used in the management of chronic granulocytic leukaemia, essential thrombocythemia, sickle cell disease, and polycythaemia vera.  ***- Chloraminophene*** is an anti-cancer chemotherapy of the alkylating family used in the treatment of certain cancers, notably leukaemia and lymphoma. It also has an immunosuppressive role.  -***2-chloro-2'-deoxyadenosine (2-CdA)*** is a chlorinated purine nucleoside with activity against lymphoproliferative disorders.  ***- Leflunomide*** is an inhibitor of pyrimidine biosynthesis and has an antiproliferative activity.  ***- Teriflunomide*** is a pyrimidine synthesis inhibitor as well, marketed as Aubagio for the treatment of multiple sclerosis. Teriflunomide is the active metabolite of leflunomide. The mechanism of action involves inhibition of dihydro-orotate dehydrogenase.  ***- Encorafenib*** is a kinase inhibitor used in combination with Benimetinib for the treatment of metastatic melanoma with the mutation V600E or V600K of *BRAF* (MIM*****164757). | ***Cisplatin:***  Decreased pressure  ***2-CdA***  Disturbance of p53 tumor suppressor pathway |
| **2.1.2 Alkylating agent** | ***- Busulfan is 1,4-butanediol dimethanesulfonate***, an alkylating agent with particular toxicity for the bone marrow. Busulfan has been used in the treatment of leukaemia and in preparation of patients for bone marrow transplantation. In addition to marrow toxicity, busulfan damaged mouse and human embryonic cells, probably through irreversible inhibition of DNA synthesis.  ***- Melphalan*** is a nitrogen mustard alkylating agent used in the treatment of some cancers. | DNA damage, Cell cycle arrest and apoptosis, induction of oxidative stress |
| **2.1.3 Anti-angiogenic agents** | Agents specifically targeting the vasculature are finding increasing use in medical practice, mainly in the treatment of colon cancer and age-related macular degeneration (8). | Effects on placental function, inhibition of angiogenesis |
|  | |  |
| **2.2 Azilsartan** | Azilsartan is an angiotensin receptor blocker (ARB) approved for the treatment of hyper­tension. ARBs are renoprotective in type 2 diabetes mellitus and may be considered as drugs of choice for renoprotection in diabetic patients. | Disruption of the Renin-Angiotensin system, reduction of blood flow |
|  | |  |
| **2.3 Treatment of Parkinson disease** | ***- Levodopa*** is an intermediary in endogenous catecholamine synthesis, used in the treatment of Parkinson disease. Levodopa exerts its effects primarily through conversion to dopamine in the central nervous system.  ***- Entacapone*** is a catechol-O-methyltransferase (COMT) inhibitor marketed as Comtan for treatment of Parkinson disease. | Unknown |
|  | |  |
| **2.4 Glucagon** | Glucagon is a 29-amino acid polypeptide hormone produced by the alpha cells of the endocrine pancreas that maintains blood glu­cose concentrations at physiological levels in the absence of exogenous carbohydrate by stimulating hepatic gluconeogenesis and glycogenolysis. Glucagon is used to treat severe hypoglycemia when the dia­betic patient cannot safely consume oral glucose and intravenous glucose is not available. | Unknown |
|  | |  |
| **2.5** **Norepinephrine** | Norepinephrine (noradrenaline) is an alpha-1 and alpha-2 adrenergic agonist used to treat hypotension. | Vasoconstrictive effects possibly leading to reduced uterine blood flow, fetal hypoxia, placental insufficiency, and/or cardiovascular Effects |
|  | |  |
| **2.6 Dexrazoxane** | Dexrazoxane is a cyclic derivative of EDTA. Razoxane is used as an immunosuppressant and cell-cycle inhibitor. Dexrazoxane is marketed for cardioprotection in patients receiving anthracycline chemotherapy. | Unknown |
|  | |  |
| **2.7 Anti-infectious** | ***- Efavirenz*** is a nonnucleoside reverse transcriptase inhibitor marketed for the treatment of HIV infection.  ***- Rifapentine*** is a rifamycin antimicrobial for the treatment of tuberculosis. It enters bacilli and binds to the β subunit of DNA-dependent RNA polymerase to form a stable drug-enzyme complex. Drug binding suppresses chain formation in RNA synthesis.  ***- Oteseconazole*** is an oral azole antifungal used for recurrent vulvovaginal candidiasis. | Unknown |

**References**

1. Somers GF. Pharmacological properties of thalidomide (alpha-phthalimido glutarimide), a new sedative hypnotic drug. Br J Pharmacol Chemother. 1960;15:111-6.

2. Patrick KS. Goodman and Gilman’s the Pharmacological Basis of Therapeutics. . Journal of Medicinal Chemistry. 2002;10th Edition Edited by J. G. Hardman, L. E. Limbird, and A. G. Gilman.:45, 6, 1392–3.

3. Tanumihardjo SA. Vitamin A: biomarkers of nutrition for development. Am J Clin Nutr. 2011;94(2):658S-65S.

4. Wadworth AN, Faulds D. Hydroxyethylrutosides. A review of its pharmacology, and therapeutic efficacy in venous insufficiency and related disorders. Drugs. 1992;44(6):1013-32.

5. Brown R, Kraus C, Fleming M, Reddy S. Methadone: applied pharmacology and use as adjunctive treatment in chronic pain. Postgrad Med J. 2004;80(949):654-9.

6. Volkow ND, Wargo EM. Overdose Prevention Through Medical Treatment of Opioid Use Disorders. Ann Intern Med. 2018;169(3):190-2.

7. Brunton L LJ, Parker K. Goodman & Gilman's The Pharmacological Basis of Therapeutics. 11th ed ed2006. 1984 p.

8. Rutland CS, Jiang K, Soff GA, Mitchell CA. Maternal administration of anti-angiogenic agents, TNP-470 and Angiostatin4.5, induces fetal microphthalmia. Mol Vis. 2009;15:1260-9.
